# Epigenomic Profiling Positions ATF7 as a Core Regulator of Colonic Inflammation

**DOI:** 10.1111/jcmm.70831

**Published:** 2025-09-03

**Authors:** Fang Liu, Yidong Chen, Jiamin Li, Junrong Li, Qi Yu, Xiaopeng Zhang, Liangru Zhu

**Affiliations:** ^1^ Division of Gastroenterology, Union Hospital, Tongji Medical College Huazhong University of Science and Technology Wuhan China; ^2^ Department of Gastroenterology The First Affiliated Hospital of Shihezi University Shihezi China; ^3^ Department of Endoscopy and Digestive System Guizhou Provincial People's Hospital Guiyang China

**Keywords:** ATF7, mitophagy, PINK1, ulcerative colitis

## Abstract

Mitochondrial dysfunction plays a central role in epithelial damage and persistent inflammation in ulcerative colitis (UC), but the transcriptional mechanisms that govern mitochondrial quality control in the intestinal epithelium remain poorly defined. Here, we identify Activating Transcription Factor 7 (ATF7) as a key regulator of mitophagy in colonic epithelial cells. Integrative transcriptomic and epigenomic analyses of patient‐derived mucosal samples revealed marked ATF7 downregulation and widespread activation of inflammatory pathways. Chromatin immunoprecipitation and luciferase reporter assays demonstrated that ATF7 directly binds to and activates the promoter of PINK1, a master regulator of mitophagy. Genetic ablation of ATF7 or PINK1 in human epithelial cells impaired mitophagy, disrupted mitochondrial membrane potential, and increased reactive oxygen species. In vivo, intestinal epithelial cell‐specific knockout of ATF7 or PINK1 exacerbated dextran sulfate sodium‐induced colitis, with greater epithelial injury, elevated cytokine production, and transcriptional activation of TNF, NF‐kappaB, and inflammatory bowel disease signalling pathways. These results establish ATF7 as a critical transcriptional regulator linking mitochondrial homeostasis to epithelial resilience in the inflamed colon.

## 1 Introduction

Ulcerative colitis (UC) is a chronic inflammatory disease of the colon characterised by persistent mucosal inflammation and recurrent episodes of abdominal pain, diarrhoea, rectal bleeding, and weight loss. Although considerable progress has been made in elucidating the pathogenesis of UC, the precise molecular mechanisms underlying its initiation and perpetuation remain incompletely understood [1, 2, 3]. Current therapeutic approaches for UC primarily focus on suppressing inflammation through immunosuppressive and biologic agents [4, 5]. However, these treatments often provide incomplete relief, fail to maintain long‐term remission, and are associated with substantial adverse effects and risks of complications. This underscores the urgent need to identify alternative and complementary therapeutic targets, particularly pathways that could restore intestinal barrier integrity and resolve chronic inflammation through mechanisms distinct from broad immunosuppression [6, 7].

Mitochondria play fundamental roles in energy metabolism, regulation of apoptosis and cellular signalling, positioning them as crucial regulators of intestinal epithelial cell integrity, inflammation, and tissue repair [8, 9, 10]. Accumulating evidence demonstrates that mitochondrial dysfunction contributes significantly to the pathophysiology of inflammatory bowel diseases, including UC, by driving epithelial barrier dysfunction, amplifying oxidative stress, and promoting chronic inflammatory responses [11, 12]. Impaired mitochondrial function not only results in energy depletion but also leads to excessive generation of reactive oxygen species (ROS), thereby exacerbating mucosal injury and inflammation. A critical mechanism by which cells mitigate mitochondrial damage is mitophagy, a selective form of autophagy specifically targeting dysfunctional mitochondria for degradation [13, 14]. Through the timely removal of damaged mitochondria, mitophagy preserves mitochondrial homeostasis, prevents oxidative injury, and maintains epithelial barrier function. Defects in mitophagy pathways are increasingly recognised to contribute directly to the chronic inflammation and mucosal damage characteristic of UC. Nevertheless, the transcriptional mechanisms that regulate mitophagy and mitochondrial homeostasis in the intestinal epithelium, especially during inflammation, remain poorly understood.

Mitophagy safeguards mitochondrial integrity by selectively eliminating dysfunctional organelles, thereby preserving cellular fitness under stress [15]. This quality control mechanism operates through distinct pathways, including BNIP3/BNIP3L, FUNDC1, and the canonical PINK1–Parkin cascade [16, 17]. BNIP3 family proteins mediate mitochondrial sequestration into autophagosomes, while FUNDC1 facilitates hypoxia‐driven turnover through direct LC3 binding [18]. The PINK1–Parkin axis serves as a central sensor of mitochondrial damage. Upon membrane depolarisation, PINK1 accumulates on the outer mitochondrial membrane, phosphorylates ubiquitin and Parkin at Ser65, and triggers Parkin's E3 ligase activity [19, 20, 21]. The ensuing ubiquitylation of mitochondrial surface proteins, primarily through K63‐ and K11‐linked chains, establishes docking platforms for autophagy receptors such as p62, OPTN, and LC3, guiding the damaged organelles toward lysosomal degradation [22]. This surveillance system is critical for controlling oxidative stress and maintaining metabolic balance. However, the contribution of PINK1–Parkin–driven mitophagy to intestinal epithelial homeostasis, particularly during inflammation‐induced epithelial injury in ulcerative colitis, remains incompletely understood. Delineating this pathway's role in mucosal defence may uncover novel targets for restoring epithelial resilience in chronic colitis.

Among various transcriptional regulators implicated in inflammation and stress responses, members of the Activating Transcription Factor (ATF) family have garnered particular attention due to their broad regulatory influence on cell survival, metabolism, inflammation, and cellular stress responses [23, 24, 25, 26]. The ATF family, which belongs to the basic leucine zipper (bZIP) superfamily of transcription factors, modulates gene expression by binding specifically to consensus cAMP response elements (CREs) present within target gene promoters. These transcription factors have diverse and context‐dependent roles in mediating cellular adaptations to environmental stress, metabolic challenges, and inflammatory stimuli [27, 28, 29, 30]. Notably, emerging evidence indicates that ATF7, a key member of this family, exerts critical roles in cellular homeostasis by governing responses to inflammatory stress, oxidative injury, and metabolic perturbations [31, 32, 33]. Although ATF7 has been studied extensively in contexts such as cardiovascular diseases, cancer, and metabolic disorders, its role in gastrointestinal inflammation, particularly UC, has not yet been systematically explored.

In light of these critical knowledge gaps, our study aims to elucidate the role of ATF7 as a potential regulator of mitochondrial quality control and inflammatory signalling in UC. Specifically, we hypothesised that ATF7 transcriptionally governs key components of the mitochondrial quality control machinery, thereby safeguarding intestinal epithelial cells against inflammation‐induced mitochondrial injury. To test this hypothesis, we performed comprehensive transcriptomic analyses of intestinal mucosa from UC patients and healthy controls, revealing significant downregulation of ATF7 expression during active disease phases. Furthermore, integrated epigenomic and functional analyses, including chromatin immunoprecipitation sequencing (ChIP‐seq) and dual‐luciferase assays, were employed to investigate direct transcriptional targets of ATF7 in epithelial cells, identifying PINK1 as a critical mitophagy‐related target gene. Using intestinal epithelial‐specific knockout mouse models, we further validated these findings in vivo by demonstrating that ATF7 deficiency significantly impairs mitochondrial integrity, exacerbates ROS accumulation, and worsens intestinal inflammation in response to DSS‐induced colitis. These results collectively establish ATF7 as a critical transcriptional hub linking mitochondrial homeostasis with inflammatory signalling in UC.

By providing novel insights into the interplay between transcriptional regulation, mitochondrial function, and epithelial inflammation, our findings not only deepen our understanding of UC pathogenesis but also offer innovative therapeutic possibilities aimed at enhancing mitochondrial integrity as a complementary or alternative approach to conventional immunosuppressive treatments.

## 2 Methods

### 2.1 Human Colonic Biopsies for Epigenomic and Transcriptomic Profiling

Colonic mucosal samples were obtained from six patients with active ulcerative colitis (UC) and six non‐inflammatory, non‐neoplastic controls undergoing diagnostic colonoscopy. UC diagnosis was confirmed clinically, and disease severity was assessed using the Mayo endoscopic subscore (MES 2–3) in conjunction with symptoms such as abdominal pain, diarrhoea, and mucopurulent bloody stool. Biopsies were flash‐frozen in liquid nitrogen and stored at −80°C for ChIP‐seq and RNA‐seq analyses. The study was approved by the Ethics Committee of Union Hospital, Tongji Medical College, Huazhong University of Science and Technology, and conducted in accordance with the Declaration of Helsinki.

### 2.2 IEC‐Specific Knockout Mouse Models and Colitis Induction

C57BL/6J wild‐type (WT) mice were obtained from Cavens (Changzhou, China). To generate intestinal epithelial cell‐specific knockouts, *Atf7* ^flox/flox^ and *Pink1* ^flox/flox^ mice (Modelorg, Shanghai; Cyagen, Guangzhou) were crossed with Villin‐Cre mice (Jackson Laboratory, Bar Harbour, ME), all on a C57BL/6J background, yielding *Atf7* ^ΔIEC^ and *Pink1* ^ΔIEC^ strains, hereafter referred to as *ATF7* ^*−/−*^ and *PINK1* ^*−/−*^, respectively. All genetically modified lines were generated using CRISPR/Cas9 technology.

Mice were housed under standard conditions with ad libitum access to chow and water. Acute colitis was induced by administering 2.5% (w/v) dextran sulfate sodium (DSS; MP Biomedicals, Cat# 9011, CA, USA) in drinking water for 8 days. On day 9, mice (*n* = 6 per group) were euthanized, and distal colonic tissues were harvested. A 0.5‐cm segment was fixed for histological analyses, including haematoxylin and eosin (H&E) staining. All animal procedures were approved by the Institutional Animal Care and Use Committee of Tongji Medical College, Huazhong University of Science and Technology.

### 2.3 Extraction of Colonic Epithelial Cells for Transcriptomic and Proteomic Profiling

Colonic epithelial cells were isolated from mouse tissue using a chelation‐based dissociation protocol [34]. Briefly, colons were excised, rinsed thoroughly with ice‐cold DPBS, and sectioned into 0.5‐cm fragments. Tissues were incubated at 4°C for 75 min in EDTA/DTT buffer (14 mL DPBS, 0.9 mL 0.5 M EDTA, 22.5 μL 1 M DTT) with gentle agitation to promote epithelial detachment. Following incubation, samples were subjected to three cycles of centrifugation and resuspension in fresh EDTA/DTT buffer to enrich for epithelial cells. After each centrifugation step, supernatants were discarded and pellets were resuspended in 5 mL of buffer. The final cell suspension was filtered through a 40‐μm mesh to remove debris and obtain a single‐cell epithelial fraction. Isolated IECs were immediately processed for downstream applications, including quantitative PCR, Western blotting, and bulk RNA sequencing.

### 2.4 ATF7 ChIP‐Seq in Ulcerative Colitis Mucosa

Chromatin immunoprecipitation sequencing (ChIP‐seq) was performed on intestinal mucosal samples from UC patients to map ATF7 binding sites. Tissues were cross‐linked, and nuclear lysates were prepared and sonicated to shear chromatin. DNA‐protein complexes were immunoprecipitated using the Simple ChIP Plus Enzymatic Chromatin IP Kit (Cell Signalling Technology, Cat# 9004, MA, USA) and anti‐ATF7 antibody (Abcam, Cat# ab183507, Cambridge, UK). Sequencing was conducted at Frasergen (Wuhan, China). Raw reads were quality‐filtered and aligned to the human reference genome (hg38), and peaks were called to identify genome‐wide ATF7 occupancy. Peak regions were visualised using the Integrative Genomics Viewer (IGV).

### 2.5 RNA Sequencing and Transcriptomic Analysis

Colonic tissues from UC patients and controls, as well as from *ATF7* ^*−/−*^ + DSS, *PINK1* ^*−/−*^ + DSS, and their respective WT + DSS mouse groups (*n* = 3 per group), were subjected to RNA sequencing. RNA extraction, quality assessment, and sequencing were performed by Cosmos Wisdom Co. Ltd. (Hangzhou, China) using the Illumina NovaSeq 6000 platform. Differential expression analysis was conducted with the DESeq2 package in R, applying the Benjamini–Hochberg method to control the false discovery rate. Genes with |fold change| > 2 and *p* < 0.05 were considered significantly differentially expressed. Data visualisation was performed using ggplot2 and ComplexHeatmap. Functional enrichment analyses, including Gene Ontology (GO), KEGG pathway analysis, and Gene Set Enrichment Analysis (GSEA), were carried out using a significance threshold of *p* < 0.05.

### 2.6 Histological Staining and Analysis

Distal colon segments (0.5 cm) were collected from euthanized mice, fixed in 10% neutral‐buffered formalin overnight at room temperature, embedded in paraffin, and sectioned at 5 μm. Sections were stained with haematoxylin and eosin (H&E) following standard protocols. Histopathological scoring of inflammation, epithelial damage, and immune cell infiltration was performed independently by two blinded pathologists; discrepancies were resolved by consensus.

### 2.7 Cell Culture and CRISPR‐Mediated Gene Editing

The human colonic epithelial cell line CCD841 CoN (ATCC, Manassas, VA) was cultured according to the supplier's instructions. ATF7‐ and PINK1‐deficient cells were generated using CRISPR/Cas9 gene editing. Single guide RNAs (sgRNAs) targeting ATF7 or PINK1 were synthesised and delivered by Genechem (Shanghai, China). Cells were transfected with sgRNA/Cas9 constructs using Lipofectamine 3000 (Thermo Fisher Scientific) following the manufacturer's protocol.

### 2.8 Cytokine Quantification in Colonic Tissue

Cytokine levels in mouse colon homogenates were measured by ELISA following the manufacturers' protocols. Kits for IL‐1β (Proteintech, Cat# KE00021, Wuhan, China), TNF‐α (Beyotime, Cat# PT513, Shanghai, China), and IL‐17 (Beyotime, Cat# PI545, Shanghai, China) were used. Absorbance at 450 nm was recorded using an Enspire microplate reader (PerkinElmer, Waltham, MA, USA). Cytokine concentrations were calculated from standard curves, and all samples were assayed in triplicate.

### 2.9 Gene Expression Analysis By qPCR

Quantitative real‐time PCR was performed using a LightCycler system (Roche Diagnostics) and SYBR Green PCR Master Mix (Takara, Cat# RR036A). Each reaction contained cDNA, gene‐specific primers (sourced from PrimerBank: https://pga.mgh.harvard.edu/primerbank/), and SYBR Green reagent. Thermal cycling conditions were as follows: 95°C for 3 min, followed by 40 cycles of 95°C for 15 s, 60°C for 30 s, and 72°C for 30 s. Amplification specificity was confirmed by melting curve analysis. All reactions were performed in triplicate, and relative expression levels were normalised to GAPDH.

### 2.10 Flow Cytometric Analysis of Mitochondrial Membrane Potential and ROS

Mitochondrial membrane potential (Δψm) and intracellular reactive oxygen species (ROS) levels were assessed in CCD841 CoN cells by flow cytometry. Δψm was measured using the Mitochondrial Membrane Potential Detection Kit (BD Biosciences, Cat# 551302), and cells were incubated with JC‐1 dye at 37°C for 30 min. Red fluorescence (590 nm) indicates intact Δψm, while green fluorescence (527 nm) reflects Δψm loss. ROS production was evaluated using 2′,7′‐dichlorofluorescein diacetate (DCFH‐DA; Sigma‐Aldrich, Cat# 35845) under identical staining conditions. Flow cytometry was performed on a BD FACSCalibur system (BD Biosciences), acquiring a minimum of 10,000 events per sample. Data were analysed with FlowJo software (Tree Star).

### 2.11 Immunoblotting of Colonic Epithelial Proteins

IECs were isolated from murine colonic tissue, and proteins were extracted using RIPA buffer (Thermo Fisher Scientific) supplemented with protease and phosphatase inhibitors (Roche). Protein concentrations were determined by BCA assay (Pierce), and equal amounts (20–30 μg) were resolved by SDS‐PAGE (10%–12%) and transferred to PVDF membranes (Millipore). Membranes were blocked with 5% non‐fat milk or BSA in TBS‐T, incubated overnight at 4°C with primary antibodies, and probed with HRP‐conjugated secondary antibodies. Signals were visualised by enhanced chemiluminescence (GE Healthcare) and quantified using ImageJ (NIH). Primary antibodies included anti‐GAPDH (Cell Signalling Technology, 5174S), anti‐PINK1 (Abcam, ab216144), anti‐ATF7 (Abcam, ab183507), and anti‐LC3B (Sigma‐Aldrich, L7543). GAPDH served as a loading control.

### 2.12 ATF7 ChIP Analysis of Mitophagy Gene Promoters

Chromatin immunoprecipitation (ChIP) assays were performed in CCD 841 CoN cells to assess ATF7 occupancy at the promoter regions of mitophagy‐related genes, including PINK1, BNIP3, BNIP3L, FUNDC1, and PRKN. Cells were cross‐linked with formaldehyde, and chromatin was isolated and digested using the SimpleChIP Plus Enzymatic Chromatin IP Kit (Cell Signalling Technology, Cat# 9004, MA, USA) according to the manufacturer's protocol. Lysates were incubated overnight at 4°C with anti‐ATF7 antibody (Abcam, Cat# ab183507, Cambridge, UK) or normal rabbit IgG as a negative control. Immune complexes were captured with magnetic beads for 2 h at 4°C with gentle rotation. Bound DNA was eluted, purified, and analysed by PCR using primers designed with Primer6 software.

### 2.13 PINK1 Promoter Activity Assessed By Dual‐Luciferase Reporter Assay

Promoter activity of PINK1 was assessed using luciferase reporter constructs based on the pGL4.11‐Basic vector (Promega, WI, USA) containing either wild‐type or mutant promoter sequences. ATF7‐knockdown and control CCD841 CoN cells were co‐transfected with reporter constructs and the pRL Renilla luciferase vector (Promega) as an internal control. Dual‐luciferase activity was measured using a luminometer (Promega, Madison, USA). Firefly luciferase signals were normalised to Renilla luciferase to account for transfection efficiency. Normalised Firefly activity was used to quantify promoter responsiveness under each condition.

### 2.14 Statistical Methods

Data are presented as mean ± standard deviation. Two‐tailed unpaired Student's *t* tests were used for pairwise comparisons, and one‐way ANOVA for multiple group comparisons. Statistical significance was defined as *p* < 0.05. Analyses were performed using GraphPad Prism v9.5 (GraphPad Software).

## 3 Results

### 3.1 Transcriptomic Analysis Identifies ATF7 Downregulation and Enrichment of Inflammatory Pathways in Ulcerative Colitis

**FIGURE 1:**
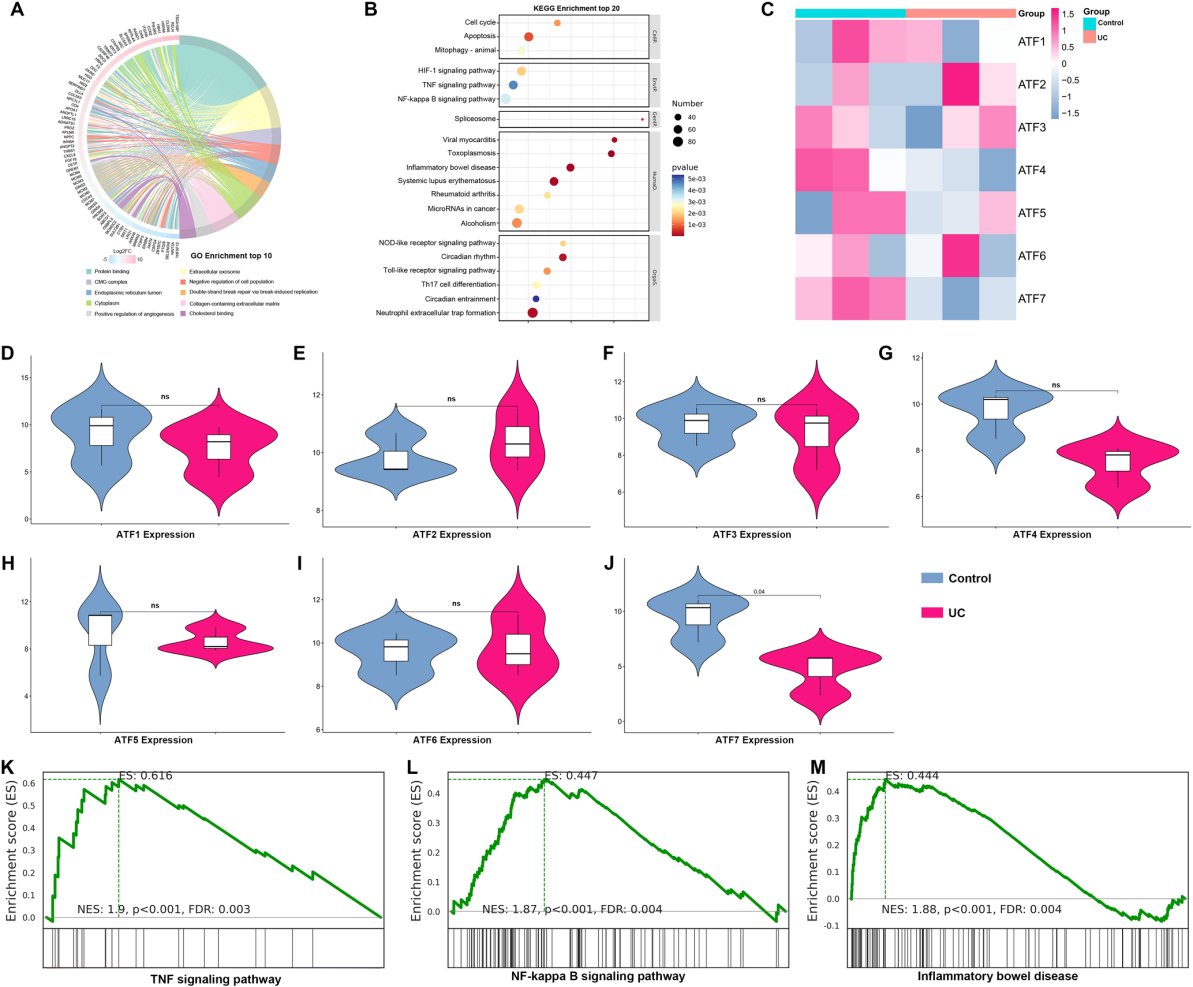
Transcriptomic profiling reveals ATF7 suppression and activation of inflammatory pathways in UC. (A) Circular chord diagram showing the top 10 enriched Gene Ontology (GO) terms among differentially expressed genes (DEGs) between UC patients and non‐inflamed controls. GO terms include protein binding, extracellular matrix organisation, regulation of cell population, and subcellular localisation. (B) KEGG pathway enrichment analysis of DEGs, highlighting significant enrichment in pathways related to apoptosis, mitophagy, HIF‐1 signalling, TNF signalling, NF‐κB signalling, inflammatory bowel disease, Toll‐like and NOD‐like receptor signalling and circadian rhythm. (C) Heatmap showing relative expression of ATF1–ATF7 in control and UC samples. ATF1, ATF4, ATF5 and ATF7 exhibit decreased expression in UC. (D–J) Violin plots comparing expression levels of ATF1–ATF7 between UC and control groups. Only ATF7 shows a significant decrease in UC tissues (J), while others show no significant change (D–I). Data are presented as mean ± SD, with statistical significance determined using unpaired two‐tailed *t*‐tests. (K–M) Gene Set Enrichment Analysis (GSEA) showing significant upregulation of the TNF signalling pathway (K), NF‐κB signalling pathway (L), and inflammatory bowel disease pathway (M) in UC samples relative to controls. NES, normalised enrichment score; FDR, false discovery rate. (*n* = 3, *p* < 0.05; ns, not significant)

We performed transcriptomic profiling of intestinal mucosal samples from patients with ulcerative colitis (UC) and non‐inflamed controls to identify disease‐associated gene expression changes. Differentially expressed genes were subjected to Gene Ontology (GO) and Kyoto Encyclopedia of Genes and Genomes (KEGG) enrichment analyses. GO analysis revealed significant enrichment in terms related to protein binding, subcellular localization (including cytoplasm and endoplasmic reticulum lumen), extracellular matrix organisation, and regulation of cell population dynamics and repair processes (Figure 1A). KEGG analysis further showed that these genes were involved in pathways such as apoptosis, mitophagy, HIF‐1 signalling, TNF signalling, NF‐κB signalling, inflammatory bowel disease, NOD‐like receptor signalling, Toll‐like receptor signalling, and circadian rhythm regulation (Figure 1B), indicating broad transcriptomic reprogramming in UC characterised by inflammation, stress response, and tissue remodelling.

To identify potential upstream regulators of these transcriptomic changes, we examined the expression of the activating transcription factor (ATF) family. Heatmap analysis showed decreased expression of ATF1, ATF4, ATF5, and ATF7 in UC samples compared to controls (Figure 1C). While no significant differences were observed for ATF1 to ATF6 in individual comparisons (Figure 1D–I), ATF7 expression was significantly reduced in UC mucosa (Figure 1J), suggesting that ATF7 downregulation may be specifically associated with the inflammatory state.

Gene Set Enrichment Analysis (GSEA) supported these observations by revealing increased activity in TNF signalling, NF‐κB signalling, and the inflammatory bowel disease pathway in UC samples relative to controls (Figure 1K–M). Collectively, these findings highlight ATF7 as a selectively suppressed transcription factor in UC and suggest a role for ATF7 in modulating inflammatory and stress‐responsive signalling networks in the intestinal epithelium.

These results collectively position ATF7 as a transcription factor selectively downregulated in the inflamed mucosa of UC patients. Its suppression coincides with the activation of pro‐inflammatory and stress‐related signalling pathways, suggesting that ATF7 may function as a key epithelial‐intrinsic regulator of immune homeostasis and barrier integrity in the context of ulcerative colitis.

### 3.2 ATF7 Directly Regulates PINK1 to Control Mitophagy and Mitochondrial Stress in Ulcerative Colitis

**FIGURE 2:**
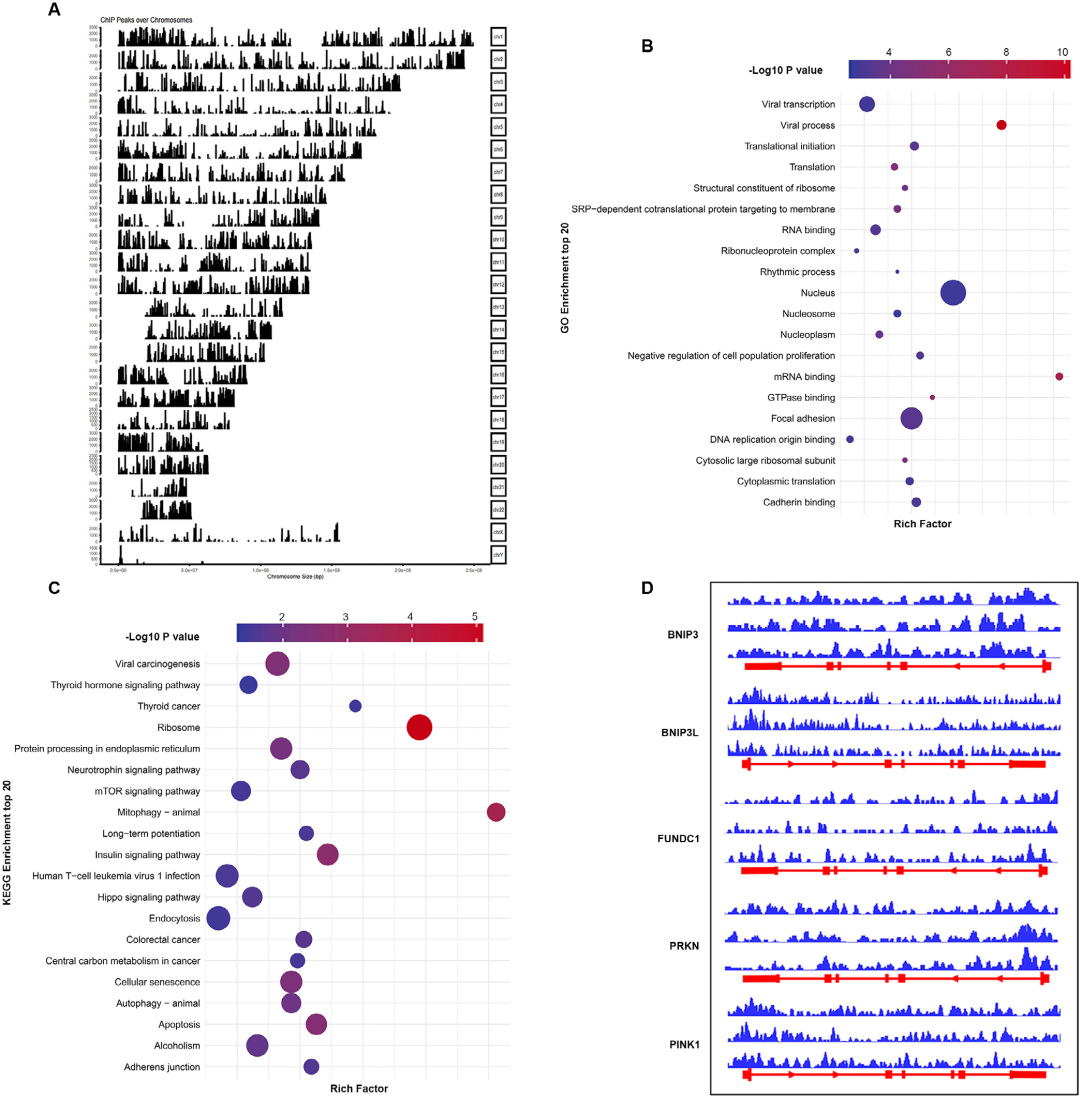
ATF7 binds to mitophagy‐related gene loci in ulcerative colitis. (A) Genome‐wide distribution of ATF7 ChIP‐seq signal peaks across human chromosomes in colonic mucosal samples from UC patients. The *x*‐axis represents chromosome length, and the *y*‐axis indicates peak intensity. (B) Gene Ontology (GO) enrichment analysis of genes near ATF7 binding sites, highlighting categories such as transcriptional regulation, RNA binding, and nucleic acid metabolism. (C) KEGG pathway enrichment analysis of ATF7 target genes. Mitophagy displayed the highest enrichment, along with pathways related to apoptosis, autophagy, mTOR signalling, and protein processing in the endoplasmic reticulum. (D) ChIP‐seq peak visualisation in the IGV browser showing ATF7 binding at the promoter regions of key mitophagy‐associated genes, including BNIP3, BNIP3L, FUNDC1, PRKN, and PINK1. These loci represent potential direct transcriptional targets of ATF7 (*n* = 3).

To investigate the direct transcriptional targets of ATF7 in UC, we performed chromatin immunoprecipitation followed by high‐throughput sequencing (ChIP‐seq) using an anti‐ATF7 antibody on colonic mucosal samples from UC patients. The genome‐wide distribution of ATF7 binding sites was visualised by plotting signal peaks across all chromosomes, revealing broad occupancy throughout the genome (Figure 2A). The *x*‐axis represents chromosome size, while the *y*‐axis reflects signal intensity, indicating ATF7 binding enrichment at specific loci.

To explore the biological relevance of ATF7 binding, we performed Gene Ontology (GO) and Kyoto Encyclopedia of Genes and Genomes (KEGG) enrichment analyses on genes proximal to ATF7 peaks. GO terms were enriched for RNA‐binding processes, transcriptional regulation, and ribonucleoprotein complex assembly (Figure 2B), suggesting a role for ATF7 in RNA metabolic and nuclear processes. KEGG pathway analysis revealed significant enrichment in mitophagy, mTOR signalling, protein processing in the endoplasmic reticulum, apoptosis, and autophagy pathways, with mitophagy displaying the highest enrichment ratio (Figure 2C), indicating a potential regulatory role of ATF7 in mitochondrial quality control.

To further explore this, we visualised ATF7 occupancy at key mitophagy‐related genes using the Integrative Genomics Viewer (IGV). Notably, ATF7 binding was observed at the promoter regions of BNIP3, BNIP3L, FUNDC1, PRKN, and PINK1, all of which are core regulators of mitophagy (Figure 2D). These results suggest that ATF7 may directly regulate genes involved in mitochondrial clearance, linking its transcriptional activity to mitochondrial homeostasis during UC pathogenesis.

**FIGURE 3:**
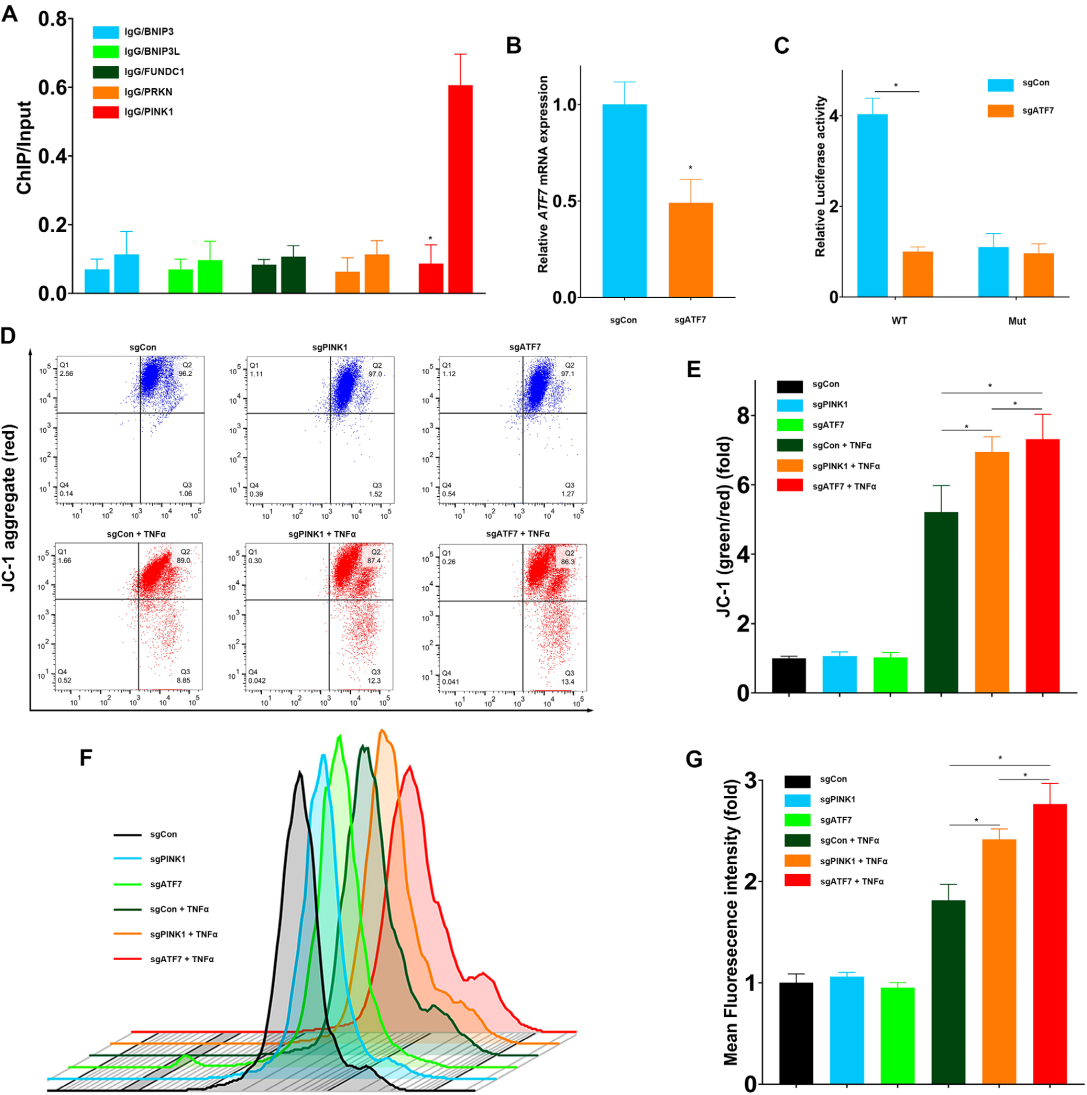
ATF7 directly regulates PINK1 transcription and maintains mitochondrial integrity under stress. (A) ChIP‐qPCR showing ATF7 binding at the promoter regions of mitophagy‐related genes. Significant enrichment was observed only at the PINK1 promoter. (B) RT‐qPCR confirming ATF7 knockdown in CCD841 cells using SgRNA. (C) Dual‐luciferase reporter assay using wild‐type (WT) and mutant (Mut) PINK1 promoter constructs. ATF7 knockdown significantly reduced luciferase activity in the WT construct but not in the mutant, indicating direct transcriptional regulation. (D) JC‐1 staining and flow cytometry showing changes in mitochondrial membrane potential (Δψm) following TNF‐α treatment. Red fluorescence (JC‐1 aggregates) decreases with PINK1 or ATF7 knockdown. (E) Quantification of the JC‐1 red/green ratio, indicating mitochondrial depolarisation in ATF7‐ or PINK1‐deficient cells. (F) ROS levels measured by flow cytometry in the presence or absence of TNF‐α. (G) Quantification of mean fluorescence intensity from ROS staining. ATF7 knockdown resulted in greater ROS accumulation than PINK1 knockdown. Data represent mean ± SD; *p* < 0.05 by Student's *t*‐test or one‐way ANOVA, *n* = 6.

To validate the transcriptional role of ATF7 in regulating mitophagy, chromatin immunoprecipitation (ChIP) assays were performed in human colorectal epithelial CCD841 cells. Among the mitophagy‐related genes assessed—BNIP3, BNIP3L, FUNDC1, PRKN, and PINK1—only PINK1 showed significant ATF7 enrichment at its promoter region, suggesting it may be a direct transcriptional target of ATF7 (Figure 3A). To functionally assess this regulatory relationship, we knocked down ATF7 using CRISPR‐Cas9‐mediated SgRNA targeting (Figure 3B). This genetic perturbation allowed us to determine whether loss of ATF7 alters PINK1 promoter activity.

To further confirm this regulatory relationship, a dual‐luciferase reporter assay was conducted using constructs containing either the wild‐type or a mutant PINK1 promoter. Silencing of ATF7 significantly reduced luciferase activity driven by the wild‐type promoter, but had no effect on the mutant construct, indicating that ATF7 directly binds and transcriptionally activates the PINK1 promoter (Figure 3C).

We next examined the impact of ATF7 loss on mitochondrial homeostasis in human colonic epithelial CCD 841 CoN cells. Mitochondrial stress was induced by TNF‐α treatment, and mitochondrial membrane potential (Δψm) was assessed by JC‐1 staining followed by flow cytometry. Knockdown of either ATF7 or PINK1 resulted in reduced Δψm, as indicated by a shift from red to green fluorescence. Notably, ATF7‐deficient cells exhibited a more pronounced mitochondrial depolarisation compared to PINK1‐deficient cells (Figure 3D,E), suggesting that ATF7 plays a critical upstream role in maintaining mitochondrial integrity under inflammatory stress.

Intracellular reactive oxygen species (ROS) levels were also evaluated by flow cytometry. TNF‐α stimulation elevated ROS production in both PINK1‐ and ATF7‐deficient cells, with the highest ROS accumulation observed in the absence of ATF7 (Figure 3F,G). These findings support a model in which ATF7 acts upstream of PINK1 to maintain mitochondrial function through transcriptional regulation of mitophagy‐related genes. In addition to regulating PINK1‐dependent mitophagy, ATF7 may influence other cellular stress responses and biological processes.

Together, these findings establish ATF7 as a critical transcriptional regulator of PINK1 and a key modulator of mitochondrial quality control in intestinal epithelial cells. By maintaining mitochondrial membrane potential and limiting ROS accumulation under inflammatory stress, ATF7 safeguards epithelial integrity. These data position ATF7 as an upstream orchestrator of mitophagy and suggest that its dysfunction may contribute to epithelial injury and chronic inflammation in ulcerative colitis.

### 3.3 ATF7 Deficiency Exacerbates Colitis via Impaired Mitochondrial Homeostasis and Amplified Inflammatory Signalling

**FIGURE 4:**
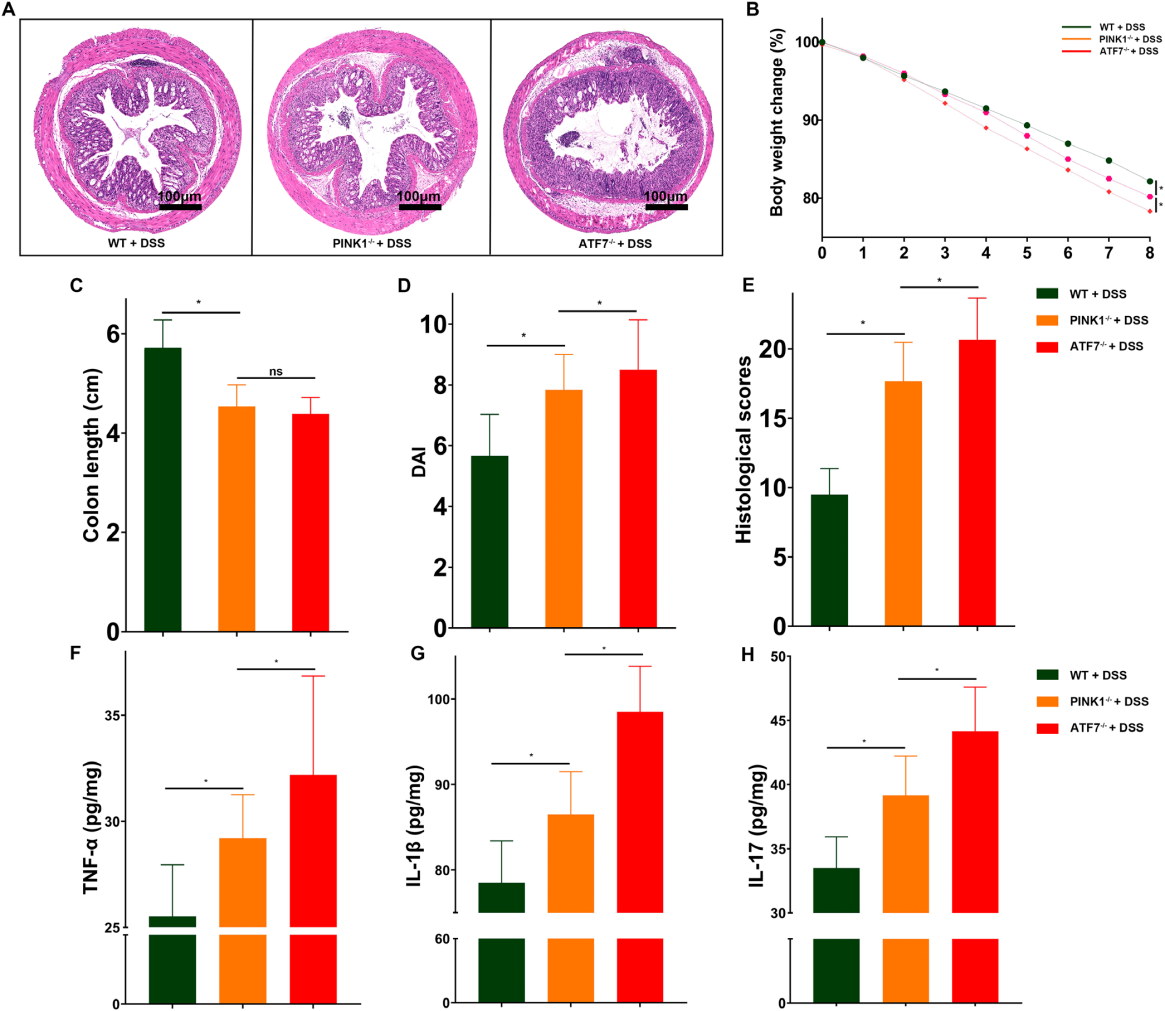
Loss of ATF7 exacerbates intestinal inflammation in DSS‐induced colitis model. (A) Representative H&E‐stained sections of distal colon tissues from WT, *PINK1* ^−/−^, and *ATF7* ^−/−^ mice following 8‐day DSS treatment. Scale bar, 100 μm. (B) Body weight change (%) during DSS administration. (C) Colon length at Day 8 post‐DSS. (D) Disease activity index (DAI) scores. (E) Histological scores based on epithelial injury and immune cell infiltration. (F–H) Quantification of colonic cytokine levels: TNF‐α (F), IL‐1β (G) and IL‐17 (H), measured by ELISA. Data are shown as mean ± SD (*n* = 6 mice per group). *p < 0.05; ns, not significant.

To assess the physiological relevance of ATF7‐mediated regulation of mitophagy in vivo, we employed DSS‐induced acute colitis models in intestinal epithelial cell‐specific ATF7‐deficient and PINK1‐deficient mice. Histological examination of colonic tissues revealed extensive epithelial disruption and immune cell infiltration in ATF7^−/−^ mice, exceeding the pathology observed in both PINK1^−/−^ and wild‐type controls (Figure 4A). During disease progression, ATF7^−/−^ mice exhibited greater weight loss (Figure 4B), more pronounced colonic shortening (Figure 4C), elevated disease activity index scores (Figure 4D), and higher histopathological injury scores (Figure 4E), indicating exacerbated colitis severity upon ATF7 loss.

We next quantified key pro‐inflammatory cytokines in colonic tissues. ATF7^−/−^ mice exhibited significantly elevated levels of TNF‐α, IL‐1β and IL‐17 (Figure 4F–H), consistent with enhanced intestinal inflammation. These findings mirror in vitro observations of mitochondrial dysfunction and oxidative stress in ATF7‐deficient cells, reinforcing the notion that ATF7 plays a protective role against colonic inflammation, likely through transcriptional control of mitophagy and inflammatory signalling.

**FIGURE 5:**
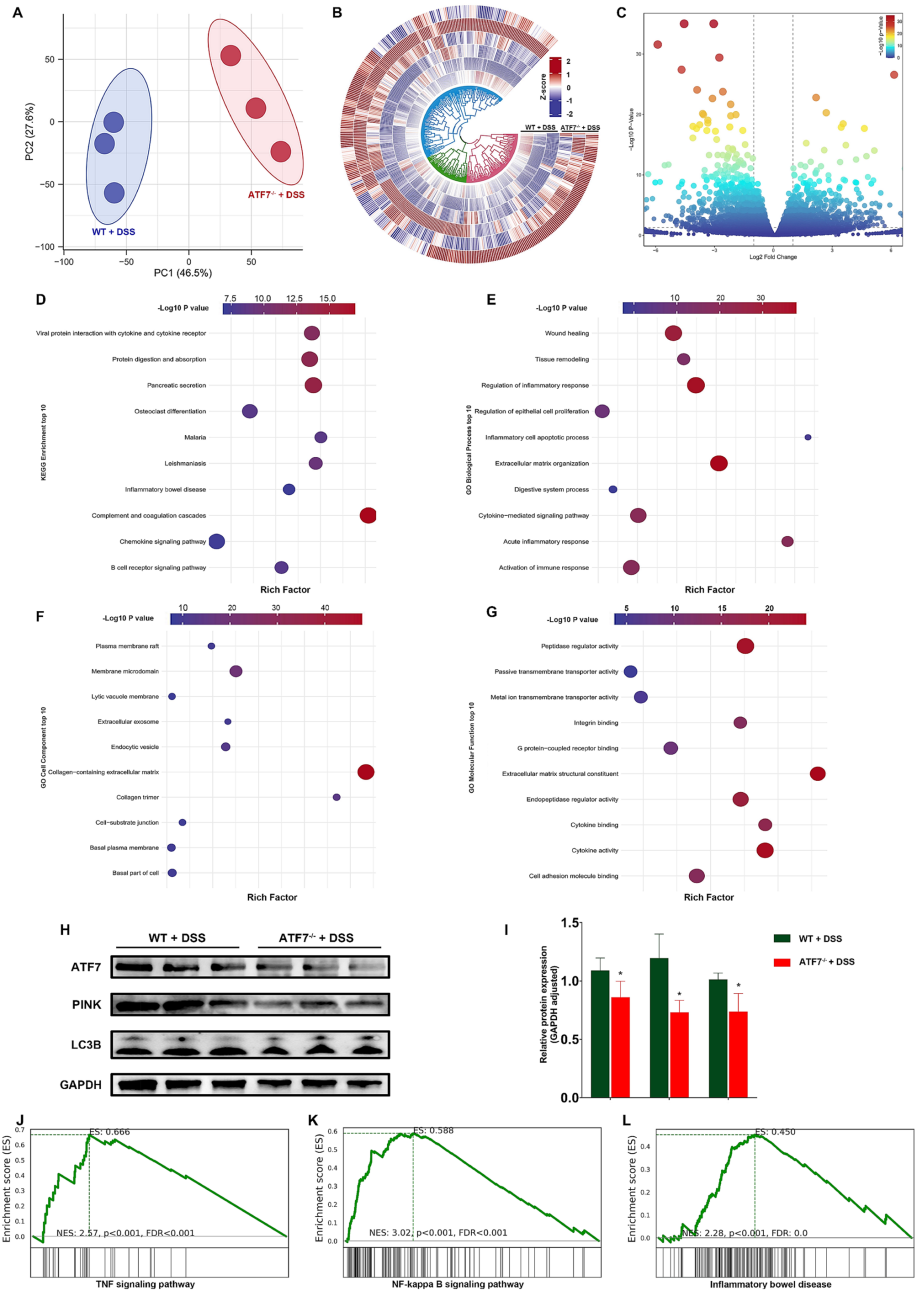
ATF7 deficiency induces transcriptomic reprogramming and suppresses mitophagy in colonic epithelium during DSS‐induced colitis. (A) Principal component analysis (PCA) of transcriptomic profiles from colonic tissues of WT + DSS and *ATF7* ^*−/−*^ + DSS mice, showing clear separation between groups. (B) Circular heatmap of differentially expressed genes, illustrating hierarchical clustering between WT + DSS and *ATF7* ^*−/−*^ + DSS samples. (C) Volcano plot of differentially expressed genes between groups. (D–G) Gene enrichment analyses of differentially expressed genes: (D) KEGG pathway enrichment, (E) GO Biological Process, (F) GO Cellular Component, and (G) GO Molecular Function. Rich factor indicates the ratio of differentially expressed genes to total genes in each term; dot size reflects gene count and colour indicates significance. (H) Representative Western blots of ATF7, PINK1, LC3B, and GAPDH in colonic tissues from WT + DSS and *ATF7* ^*−/−*^ + DSS mice. (I) Quantification of Western blot data showing reduced expression of PINK1 and LC3B in ATF7‐deficient colons. (J–L) Gene Set Enrichment Analysis (GSEA) plots show significant upregulation of (J) TNF signalling, (K) NF‐κB signalling, and (L) inflammatory bowel disease pathways in *ATF7* ^*−/−*^ + DSS colons. NES, normalised enrichment score; FDR, false discovery rate. Data are shown as mean ± SD (*n* = 3–6 mice per group). *p < 0.05; ns, not significant.

To explore the transcriptional consequences of ATF7 loss in vivo, we performed RNA sequencing on colonic tissues from wild‐type (WT + DSS) and intestinal epithelial‐specific ATF7 knockout mice (*ATF7* ^*−/−*^ + DSS) subjected to DSS‐induced colitis. Principal component analysis (PCA) revealed clear segregation between the two groups, indicating distinct transcriptional profiles (Figure 5A). Differential gene expression analysis further demonstrated substantial transcriptomic remodelling, as shown by hierarchical clustering and volcano plots (Figure 5B,C).

KEGG pathway enrichment analysis of differentially expressed genes revealed significant enrichment in immune‐related and inflammatory pathways, including B cell receptor signalling, chemokine signalling, and inflammatory bowel disease (Figure 5D). GO Biological Process terms highlighted pathways involved in extracellular matrix organisation, epithelial proliferation, cytokine signalling, and immune activation (Figure 5E). GO Cellular Component analysis identified enrichment in structures such as the plasma membrane, extracellular vesicles, and collagen‐containing matrix (Figure 5F). GO Molecular Function terms were associated with cytokine receptor binding, integrin binding, and transporter activities (Figure 5G), suggesting that ATF7 deficiency perturbs a broad array of epithelial and immunological processes.

To functionally validate the transcriptomic changes, we assessed protein levels of ATF7, PINK1, and the autophagy marker LC3B by Western blot. Consistent with transcriptional suppression, ATF7‐deficient tissues exhibited reduced PINK1 and LC3B expression (Figure 5H,I), indicating impaired mitophagy in vivo.

Finally, Gene Set Enrichment Analysis (GSEA) confirmed that the TNF signalling pathway, NF‐κB pathway, and inflammatory bowel disease pathway were significantly activated in *ATF7* ^*−/−*^ + DSS mice relative to WT + DSS controls (Figure 5J–L), reinforcing the pro‐inflammatory phenotype associated with ATF7 loss.

**FIGURE 6:**
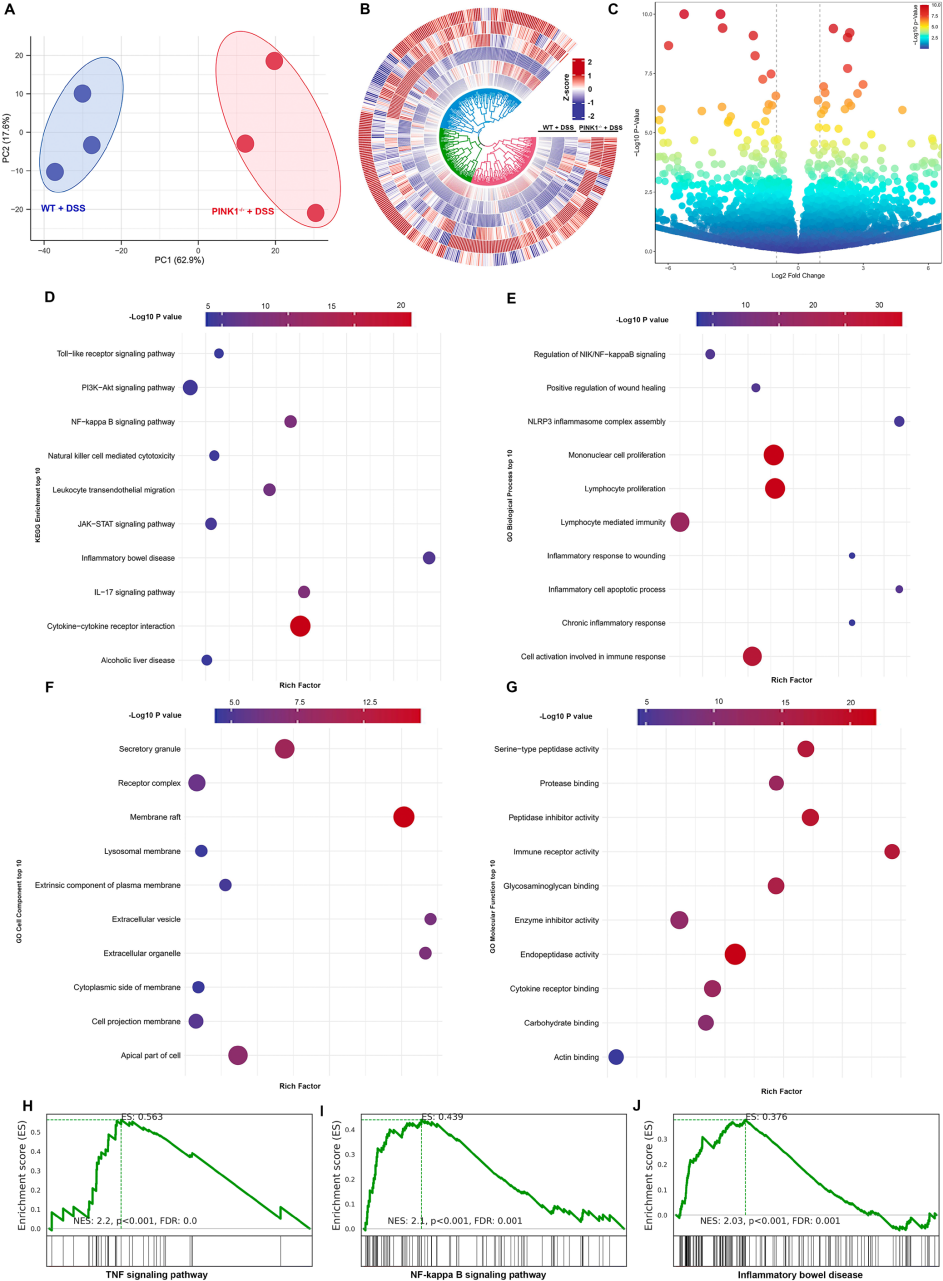
Transcriptomic Analyses Reveal Exacerbated Inflammatory Signalling in PINK1‐Deficient Mice with DSS‐Induced Colitis. (A) Principal component analysis (PCA) of RNA‐seq data from colonic tissues of WT + DSS and *PINK1* ^*−/−*^ + DSS mice demonstrates clear transcriptional separation between groups. (B) Circular heatmap depicting hierarchical clustering of differentially expressed genes between WT and PINK1‐deficient colons. (C) Volcano plot illustrating significantly upregulated (red) and downregulated (blue) genes in *PINK1* ^*−/−*^ + DSS mice compared to WT + DSS controls. (D) KEGG pathway enrichment analysis of differentially expressed genes reveals strong enrichment in immune and inflammatory pathways, including Toll‐like receptor, PI3K‐Akt, NF‐κB, IL‐17, and cytokine–cytokine receptor interaction signalling. (E–G) Gene Ontology (GO) enrichment analysis highlights altered biological processes (E), cellular components (F), and molecular functions (G) in *PINK1* ^*−/−*^ + DSS colons. Enriched GO terms include inflammatory apoptosis, lymphocyte‐mediated immunity, NLPR3 inflammasome assembly, cytokine receptor binding, and lysosomal membrane involvement. (H–J) Gene Set Enrichment Analysis (GSEA) further confirms upregulation of TNF signalling (H), NF‐κB signalling (I), and inflammatory bowel disease–associated gene programmes (J) in PINK1‐deficient colons (*n* = 3).

To delineate the transcriptomic impact of PINK1 deficiency during colitis, we performed RNA‐seq on colonic tissues from WT + DSS and *PINK1* ^*−/−*^ + DSS mice. Principal component analysis (PCA) demonstrated a clear separation between the two groups, indicating distinct gene expression profiles (Figure 6A). Hierarchical clustering of differentially expressed genes, visualised in a circular heatmap, revealed systematic transcriptional divergence between WT and PINK1‐deficient colons (Figure 6B). The volcano plot further highlighted numerous genes with significant upregulation or downregulation in the absence of PINK1 (Figure 6C).

To elucidate the biological functions of these transcriptional alterations, we conducted pathway enrichment analyses. KEGG pathway analysis identified significant enrichment in pathways associated with inflammatory signalling, including Toll‐like receptor signalling, PI3K‐Akt signalling, NF‐κB signalling, cytokine–cytokine receptor interaction, and IL‐17 signalling (Figure 6D), suggesting a broad activation of immune responses. GO Biological Process terms revealed enrichment in inflammatory cell apoptotic processes, lymphocyte‐mediated immunity, NLPR3 inflammasome assembly, and chronic inflammatory responses (Figure 6E). GO Cellular Component analysis indicated prominent involvement of extracellular vesicles, lysosomal membranes, and secretory granules (Figure 6F), while GO Molecular Function terms pointed to upregulation of immune receptor activity, cytokine receptor binding, and serine‐type peptidase activity (Figure 6G).

Consistent with these results, Gene Set Enrichment Analysis (GSEA) confirmed significant activation of the TNF signalling pathway (Figure 6H), NF‐κB signalling pathway (Figure 6I), and inflammatory bowel disease–associated transcriptional programmes (Figure 6J) in *PINK1* ^*−/−*^ + DSS tissues relative to WT controls. These findings underscore the critical role of PINK1 in restraining pro‐inflammatory signalling cascades and preserving mucosal homeostasis during colitis.

In summary, our findings identify ATF7 as a key transcriptional regulator of PINK1 and mitophagy in intestinal epithelial cells. Loss of ATF7 disrupts mitochondrial homeostasis, increases ROS production, and exacerbates mucosal inflammation in colitis. These effects are partially recapitulated in PINK1‐deficient models, highlighting the ATF7–PINK1 axis as a critical pathway in maintaining epithelial integrity during inflammatory stress.

## 4 Discussion

In this study, we focused on elucidating the roles of the Activating Transcription Factor (ATF) family in ulcerative colitis (UC). A key finding is the significant downregulation of ATF7 in the intestinal mucosa of UC patients during active disease, as revealed by RNA‐seq analyses (Figure 1). This marked suppression of ATF7 implicates its pivotal role in mucosal inflammation and the underlying pathology of UC.

To further investigate the mechanism through which ATF7 exerts its effects, we employed chromatin immunoprecipitation sequencing (ChIP‐seq) and dual‐luciferase reporter assays, uncovering novel regulatory functions of ATF7. Our findings demonstrate that ATF7 directly binds to and positively regulates the transcription of PINK1, a key mitochondrial quality‐control gene critical in mitophagy (Figure 2). Subsequent cellular experiments underscored the functional importance of ATF7 and PINK1, revealing that deficiencies in either protein resulted in impaired mitochondrial membrane potential and excessive reactive oxygen species (ROS) accumulation, indicating compromised mitochondrial integrity under inflammatory conditions (Figure 3).

To validate these observations in a physiologically relevant context, we utilised intestinal epithelial cell‐specific knockout mouse models for ATF7 and PINK1, employing the DSS‐induced colitis model. Consistent with our in vitro results, ATF7 deficiency led to significant downregulation of PINK1 and suppression of mitochondrial autophagy in vivo, as evidenced by diminished LC3B expression. Notably, mice lacking ATF7 displayed more severe colonic inflammation compared to wild‐type controls and even PINK1‐deficient counterparts, highlighting the central role of ATF7 in orchestrating mitochondrial quality control and protecting against inflammation‐driven epithelial damage (Figure 4).

RNA‐seq analysis performed on mouse intestinal tissues provided further insights into the transcriptional landscapes altered by ATF7 loss, emphasising extensive dysregulation of genes associated with immune response, stress signalling pathways, and tissue remodelling. Collectively, our data strongly suggest that ATF7 is not merely a transcription factor but rather a critical node coordinating mitochondrial quality control and epithelial inflammatory responses in the gut.

This work significantly extends our current understanding of epithelial‐specific molecular mechanisms underlying UC pathogenesis. While PINK1 has been extensively studied regarding its essential role in mitophagy, our identification of ATF7 as an upstream regulator of PINK1 expression provides novel mechanistic insight. By linking transcriptional regulation directly to mitochondrial quality control, we propose that ATF7 functions as a molecular safeguard, sustaining epithelial mitochondrial integrity under inflammatory stress.

Moreover, these findings align with and significantly advance the growing body of evidence implicating mitochondrial dysfunction as a central driver in the chronic inflammatory milieu characteristic of UC. Accumulating damaged mitochondria elevate ROS production, intensifying oxidative stress and barrier disruption. Our demonstration that ATF7 deficiency exacerbates these pathological processes in vivo further underscores the crucial importance of mitochondrial homeostasis in maintaining intestinal epithelial barrier integrity and immune homeostasis.

Previous research on ATF7 has highlighted its diverse biological functions across multiple physiological and pathological contexts. ATF7 expression can be regulated by RNA methylation mechanisms; for instance, m(5)C methylation reduction enhances ATF7 levels, subsequently downregulating targets involved in necroptosis and cardiomyocyte injury [35]. In developmental biology, ATF7 is implicated in mediating epigenetic changes induced by thermal stress, affecting early embryonic development [36]. ATF7 also responds to inflammatory stimuli, such as TNF‐alpha, influencing telomere shortening through its phosphorylation‐dependent release from telomeric regions [37]. Furthermore, ATF7 forms functional heterodimers (e.g., ATF7/JDP2) involved in modulating inflammatory pathways in acute myeloid leukaemia (AML), acting to counteract gene‐activating functions and suppress inflammatory overactivation [38]. Additionally, ATF7‐dependent epigenetic modifications have intergenerational impacts, as evidenced by changes induced by paternal dietary conditions, underscoring its broad role in environmental adaptation and inheritance [39]. Collectively, these studies position ATF7 as a multifunctional transcription factor with critical roles in stress responses, inflammation, development, and epigenetic regulation.

Given the limitations and safety concerns associated with current immunosuppressive therapies for ulcerative colitis, there remains a pressing need to explore alternative and complementary strategies aimed at mitigating chronic intestinal inflammation and reinforcing epithelial barrier integrity. Targeting epithelial‐intrinsic pathways such as mitochondrial quality control provides a promising and previously underappreciated therapeutic avenue [40, 41]. Our identification of ATF7 as a key transcriptional regulator bridging mitochondrial homeostasis and inflammatory signalling highlights the therapeutic potential of enhancing mitochondrial function. Strategies directed at restoring or augmenting ATF7‐mediated transcriptional networks may offer innovative opportunities to ameliorate epithelial damage, modulate inflammatory responses, and ultimately achieve sustained remission with fewer adverse effects. Future investigations focused on the clinical feasibility and efficacy of such mitochondria‐targeted interventions could significantly expand treatment options for UC patients.

Clinically, our study has several translational implications. Given the pivotal role of ATF7 in mitigating mitochondrial damage and subsequent inflammatory cascades, therapeutic strategies aimed at restoring or enhancing ATF7 function could provide significant clinical benefits for UC management. Currently, available therapies primarily target immune responses and often fail to induce sustained remission, underscoring the urgent need for novel interventions that directly reinforce epithelial barrier integrity [42, 43]. Targeting the ATF7‐mediated signalling pathways—particularly those governing mitochondrial quality control and epithelial cell survival—represents a promising therapeutic avenue for attenuating mucosal inflammation and promoting tissue repair. Potential strategies might include small molecule activators of ATF7, epigenetic modulators capable of restoring endogenous ATF7 expression, or mitochondrial‐targeted agents designed to mimic downstream effects of the ATF7‐PINK1 signalling cascade. Furthermore, assessing ATF7 expression in clinical biopsies might serve as a valuable biomarker, enabling personalised therapeutic strategies by identifying patients most likely to benefit from mitochondrial‐focused interventions and facilitating precise monitoring of disease activity and therapeutic efficacy. Such personalised approaches may enhance clinical outcomes by specifically targeting underlying cellular dysfunctions rather than broadly suppressing immune function, potentially reducing treatment‐associated complications and side effects.

Despite the strengths of our study, several limitations warrant careful consideration. Although intestinal epithelial cell (IEC)‐specific knockout models provide robust mechanistic insights into the epithelial‐intrinsic roles of ATF7 and PINK1, it remains possible that additional cellular compartments, such as immune cells, fibroblasts, endothelial cells, or enteric neurons, also contribute to the observed phenotype [44]. Crosstalk between IECs and other intestinal resident cells could further modulate disease progression and response to mitochondrial dysfunction [45]. Thus, future studies employing conditional knockout or cell‐specific rescue strategies in these alternative cellular compartments would help clarify the comprehensive cellular and molecular landscape influenced by ATF7 signalling in colitis. Additionally, our study utilised an acute DSS‐induced colitis model, which, while valuable for elucidating mechanisms of mucosal injury and acute inflammation, does not fully recapitulate the complex, chronic, and relapsing nature of human UC. Therefore, extending these findings into chronic inflammatory models, including genetically susceptible mouse strains, long‐term administration of inflammatory agents, and patient‐derived intestinal organoids, will be critical to strengthen translational relevance and confirm the broader clinical applicability of our mechanistic insights.

Future research should aim to delineate broader regulatory networks involving ATF7. While we clearly establish ATF7's transcriptional activation of PINK1, the possibility remains that ATF7 interacts cooperatively or competitively with additional transcriptional regulators, chromatin modifiers, or noncoding RNAs to precisely fine‐tune mitochondrial dynamics and inflammatory responses in intestinal epithelia. Genome‐wide mapping of ATF7 binding sites under various inflammatory, metabolic, and stress conditions, coupled with advanced proteomic approaches to elucidate its protein–protein interactome, will offer deeper mechanistic insights and may reveal additional therapeutic targets. Further, considering the emerging links between diet, microbiota composition, and mitochondrial function, future work investigating how environmental factors modulate ATF7 activity and its downstream targets could provide novel insights into disease susceptibility and preventive strategies.

In conclusion, our study provides compelling evidence positioning ATF7 as a central transcriptional regulator connecting mitochondrial quality control with inflammation in ulcerative colitis. Through direct modulation of PINK1 and mitophagy, ATF7 maintains mitochondrial integrity, limits oxidative stress, and protects epithelial cells from inflammatory injury. These findings not only expand our understanding of epithelial stress responses and mitochondrial pathobiology in UC but also suggest innovative therapeutic approaches to specifically target mitochondrial dysfunction and mitigate inflammation‐driven epithelial damage, potentially reshaping therapeutic paradigms in inflammatory bowel diseases.

## Author Contributions

**Fang Liu:** data curation (lead), methodology (lead), writing – original draft (lead). **Yidong Chen:** data curation (supporting). **Jiamin Li:** data curation (supporting). **Junrong Li:** data curation (supporting). **Qi Yu:** data curation (supporting). **Xiaopeng Zhang:** data curation (supporting). **Liangru Zhu:** conceptualization (lead), supervision (lead).

## Ethics Statement

Human studies were approved by the Independent Ethics Committee of Wuhan Union Hospital (Approval No. 2024‐0261) and conducted in accordance with the Declaration of Helsinki. All procedures involving animals were approved by the Animal Ethics Committee of Huazhong University of Science and Technology (Approval No. 2022‐3388).

## Conflicts of Interest

The authors declare no conflicts of interest.

## Data Availability

All data supporting the findings of this study are available from the corresponding author upon reasonable request.
